# Effect of Negative Pressure Therapy on Skin Blood Flow Responses in Scar Tissue

**DOI:** 10.7759/cureus.90436

**Published:** 2025-08-18

**Authors:** Wei-Cheng Shen, Hsu-Tang Cheng, Yih-Kuen Jan, Ben-Yi Liau, Chun-Ming Lien, Jian-Guo Bau, Wen-Hung Chao, Congo T Ching, Chi-Wen Lung

**Affiliations:** 1 Department of Creative Product Design, Asia University, Taichung, TWN; 2 Department of Surgery, Asia University Hospital, Taichung, TWN; 3 Department of Health and Kinesiology, University of Illinois Urbana-Champaign, Urbana, USA; 4 Department of Automatic Control Engineering, Feng Chia University, Taichung, TWN; 5 Department of Commercial Design and Management, National Taipei University of Business, Taoyuan, TWN; 6 Department of Agricultural Technology, National Formosa University, Huwei Township, TWN; 7 Department of Cultural Creativity and Digital Marketing, National United University, Miaoli, TWN; 8 Graduate Institute of Biomedical Engineering, National Chung Hsing University, Taichung, TWN

**Keywords:** capillaries, cupping, laser doppler, scar, tensile stresses

## Abstract

Introduction: Skin blood flow is crucial for transporting nutrients during healing and treating scarring. Negative-pressure therapy (NPT) is a promising option for enhancing skin blood flow. However, the most effective dosage of NPT for treating scars remains unclear. This study aimed to investigate the effect of different dosages of NPT on skin blood flow before and after treatment.

Methods: In this study, 36 participants with scars were recruited to assess the efficacy of various NPT interventions. NPT was administered at three different dosages: -105, -125, and -145 mmHg for 10 min. The perfusion level is defined as the amount of blood flowing through the skin (SBF). In NPT, SBF was measured before treatment (SBF-Baseline), during treatment (SBF-Application), and after treatment (SBF-Termination).

Results: SBF-Termination at -105 mmHg NPT (152.7 ± 33.7 perfusion units (PU)) showed a significantly higher SBF than at -125 mmHg NPT (77.5 ± 19.2 PU, P = 0.026) or -145 mmHg NPT (65.2 ± 12.7 PU, P = 0.012). Statistical relevance was found at -105 mmHg NPT between SBF-Application and SBF-Termination (r=0.890,P < 0.001).

Conclusion: This study indicates that a -105 mmHg NPT dosage can effectively increase SBF. Our research contributes to the emerging field of scar treatment and provides evidence-based recommendations.

## Introduction

The skin is the largest organ in the human body in terms of surface area. It protects against mechanical damage, microbial infections, ultraviolet radiation, and extreme temperatures [1]. Injuries occur when the natural structure and function of the skin are compromised, leading to the development of scars [2]. The treatment of scarring is as important as that of chronic wounds. In the developed world, 100 million people are affected by scars annually, 55 million by elective surgeries, and 25 million by trauma surgeries [3]. Scars often affect a patient’s quality of life and can cause symptoms such as itching, pain, and psychological problems [4]. Scar tissue is a disorganized extracellular matrix. Collagen fibers in scar tissue are dense and have few capillaries, compromising the microcirculation system and reducing skin blood flow (SBF) [5,6]. In scar tissue, collagen directionality is disorganized and irregular, with low homogeneity and coarseness [7]. Scar tissue contains few or no capillaries because of the compression of these collagens [8]. As scar tissue lacks pores, capillaries must transport oxygen during healing, resulting in a hypoxic environment and a lack of capillaries in the scar tissue [9].

SBF plays a crucial role in reducing pathological scars by facilitating nutrient transport through the microcirculation system [10,11], thus speeding up tissue repair by providing sufficient nourishment [11,12]. Different stages of scar healing have specific needs for skin blood flow levels [12], reflecting the unique metabolic and structural requirements of healing tissue. As the inflammation phase of wound healing advances, SBF increases, encouraging the entry of mononuclear cells and macrophages into the wound. This process supports the removal of infectious agents [13]. During the six months of proliferation after skin injury, higher SBF levels are still required to deliver oxygen, nutrients, and growth factors from endothelial cells, all of which enhance collagen production by fibroblasts [14], cellular regeneration, and the development of granulation tissue [15]. The remodeling phase typically begins six months to one year after injury, during which the extracellular matrix, including myofibroblasts and collagen molecules, is realigned and crosslinked [16].

If hypoxic scar tissue persists during the remodeling phase, hypoxia-inducible factor 1 (HIF-1) may activate to promote cell survival, inhibit apoptosis, and contribute to hypertrophic scarring [17]. An increase in SBF can increase the amount of oxygen [18]. While both hypoperfusion and excessive vascular response have been linked to pathological scarring, phase-specific increases in SBF, particularly during proliferation and remodeling, are generally associated with enhanced scar quality and structural organization.

Noninvasive treatments, such as negative-pressure therapy (NPT), are promising options for enhancing SBF [19]. Its mechanism and efficacy in increasing SBF have been clinically demonstrated [20]. By creating a localized pressure differential across the skin and subdermal tissue, NPT induces visible vasodilation and localized hyperemia [21]. This improves perfusion in scar regions, where microvascular function is often compromised due to disorganized collagen and elastic fiber accumulation [22]. Moreover, NPT has been shown to align extracellular matrix components [23] and alleviate fibrotic blockage in capillaries, further increasing SBF [24]. Clinically, NPT has been reported to improve scar thickness and pliability in certain cases, with studies exploring pressure ranges between -105 and -125 mmHg based on treatment goals [25]. It may also promote collagen fiber realignment and improve the extracellular matrix organization. By enhancing perfusion, NPT can potentially reduce hypoxia-driven fibrosis. These effects support its use in managing immature or hypertrophic scars that limit mobility, particularly during the remodeling phase of scar healing.

Although NPT has demonstrated promising results in modulating scar tissue through improved skin perfusion, the optimal therapeutic parameters remain debatable. In particular, the relationship between specific pressure levels and the regulation of SBF has not been fully clarified. A crucial component of this therapy is the increased SBF, which delivers oxygen, nutrients, and cells while removing metabolic waste [26]. It triggers endothelial cell repair of capillaries and stimulates angiogenesis in local tissues [27]. Depending on the dressing material, pressures ranging from -75 to -225 mmHg are often applied to soft tissues [28]. It is generally believed that -125 mmHg provides the most conducive environment for granulation tissue growth and SBF in the skin [29]. Borgquist et al. (2010) investigated wound-edge microvascular blood flow under different negative pressures and found that -125 mmHg significantly improved perfusion without compromising tissue integrity [28]. Zhu et al. (2021), using a rabbit radius bone defect model, demonstrated that NPT at -125 mmHg enhanced tissue regeneration and vascularization more effectively than conventional therapy [29]. These findings suggest that -125 mmHg may offer a favorable balance between mechanical stress and biological response in soft tissue and bone healing.

Monitoring SBF responses to different NPT levels may help clarify how mechanical stimulation contributes to scar remodeling and tissue oxygenation [30,31]. While higher negative pressures are associated with increased perfusion, excessive pressure could potentially impair fragile capillaries in the scar tissue. Therefore, identifying the lowest effective pressure that enhances SBF without compromising microvascular integrity is important for optimizing clinical outcomes.

Our hypothesis was that NPT enhances SBF in scar tissue and that different pressure levels induce distinct perfusion responses. This study aimed to confirm the effects of different NPT dosages on scar tissue by comparing blood flow before and after NPT application. By identifying the optimal pressure for maximizing SBF without causing vascular compromise, this study aimed to provide a physiological basis for refining clinical protocols for scar management.

## Materials and methods

**Figure 1:**
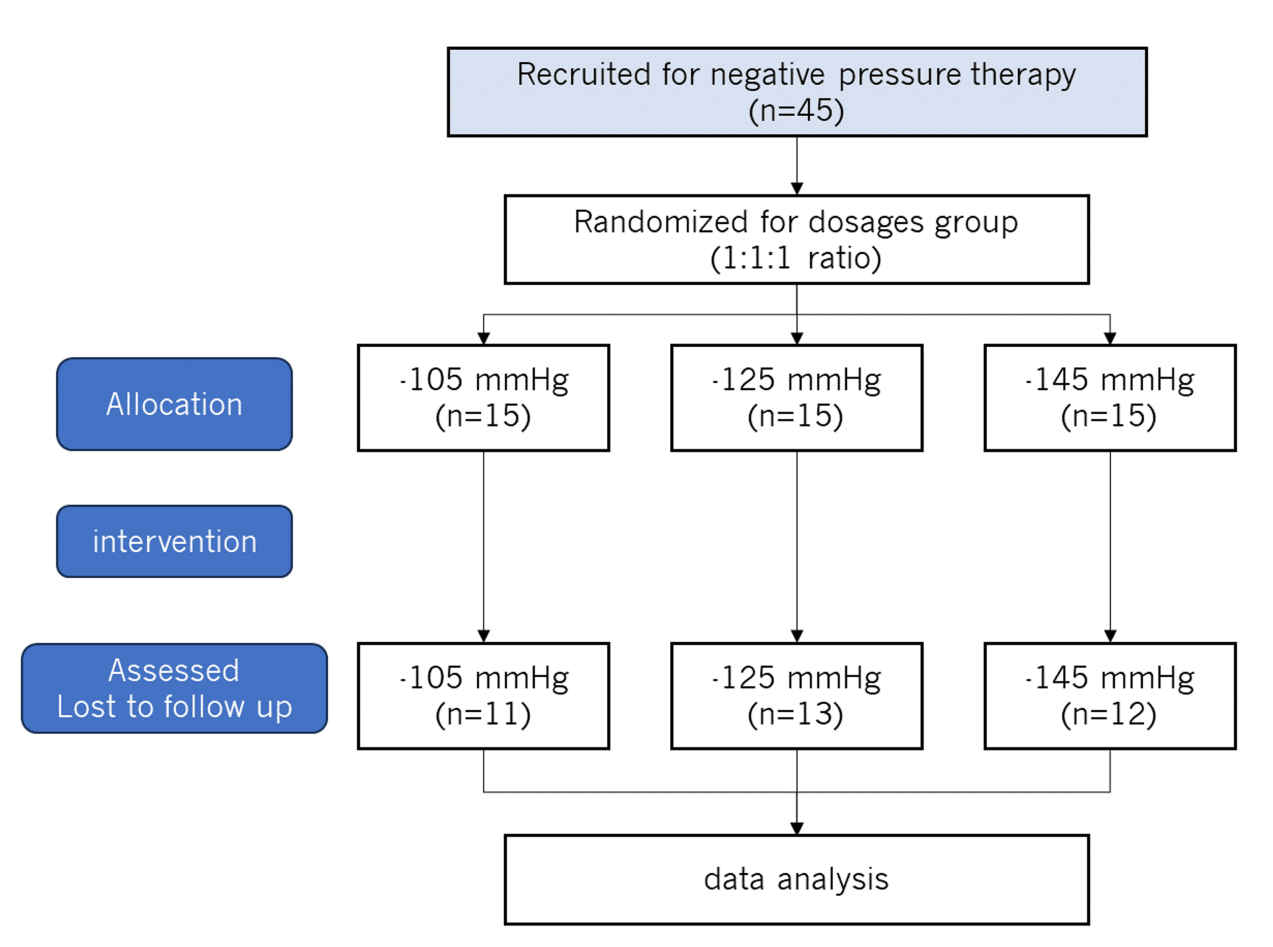
CONSORT flowchart of the study process. CONSORT: Consolidated Standards of Reporting Trials.

The study design and sample setting were drawn from patients randomly assigned to one of three parallel groups, initially in a 1:1:1 ratio, to invite participants to receive one of the NPT dosages (-105, -125, or -145 mmHg). Figure 1 displays the Consolidated Standards of Reporting Trials (CONSORT) flowchart of the assessments.

Subjects

The recruitment for this study focused on individuals with hypertrophic scars, as assessed by physicians at Asia University Hospital. Recruitment for this study began on May 11, 2022, and ended on May 11, 2023. Most subjects were in the proliferative remodeling phase. All recruited participants were approached to participate in a double-blind, parallel-group study that investigated the effects of NPT on scar healing. The room temperature was maintained at 24 ± 2°C throughout the experiment. The examinations were performed as described in our previous study [25]. Before the screening and experimental procedures, the participants signed an informed consent form approved by the Central Regional Research Ethics Committee of Chinese Medical University, Taichung, Taiwan (approval number: CRREC-111-017; approval date: May 11, 2022). This study was registered in the International Trial Registry (ClinicalTrials.gov: Identifier NCT06690749). The studies were conducted in accordance with local legislation and institutional requirements.

The inclusion criteria specified that participants were adults aged 18 years or older with scars in the proliferative or remodeling phase (within nine months of skin injury). Additionally, wounds had to be present for at least 21 days to ensure the study focused solely on scar tissue. Participants exhibiting tissue fluid leakage or edema, which may indicate abnormal capillary responses, were excluded [32]. They were in the proliferation or remodeling phase within nine months of the skin injury. The patient received long-term treatment with silicone gel, a standard conservative approach for scar management, which has minimal immediate effects on SBF. Only patients who had been consistently using silicone gel before the study were included to minimize variability. Other short-term treatments, such as pressure garments, radiotherapy, steroids, and cryotherapy, were not used to prevent the influence of different treatments on scar SBF [33]. The exclusion criteria were incomplete wound healing (leakage of tissue fluid) and edema, which may indicate an abnormal capillary mechanism [34].

Equipment and procedure

We designed the experiment based on current clinical wound NPT medical cases and experiments. Three negative-pressure changes at -105, -125, and -145 mmHg at 10 min were used in this study. A dosage of -125 mmHg was selected based on the findings of a previous study [28]. Creating an optimal environment for granulation tissue growth is crucial for wound healing [29]. In addition, to prevent damage to new capillaries, the study utilized a base dosage of -125 mmHg and avoided negative-pressure magnitudes greater than -150 mmHg [35]. We based our study on similar studies that used increments and decrements of 20 mmHg [36].

The duration of cupping therapy in this study was 10 min, which is considered a short-term effective duration for NPT [37]. The 10‑minute duration was selected based on clinical practice and safety considerations, aiming to increase the SBF without causing microvascular damage. The study suggests that continuous high-pressure NPT may decrease perfusion in poorly vascularized tissues, while shorter or intermittent applications help prevent such hypoperfusion. Therefore, our protocol uses a relatively short 10‑minute duration across all pressure groups to balance effectiveness with the reduction of capillary stress or rupture in scar tissue (Borgquist et al., 2010) [28]. The SBF signals were recorded and sampled at 40 Hz. A laser Doppler flowmeter (MoorVMS-LDF, Moor Instruments Ltd., Axminster, United Kingdom) was used to measure the changes in SBF [38]. Through the Doppler effect, the monitor receives the laser beams scattered by the moving red blood cells, which were screened out, and the signal was processed, calculated, and converted into readable digital data, which were mainly used to detect subcutaneous SBF.

**Figure 2:**
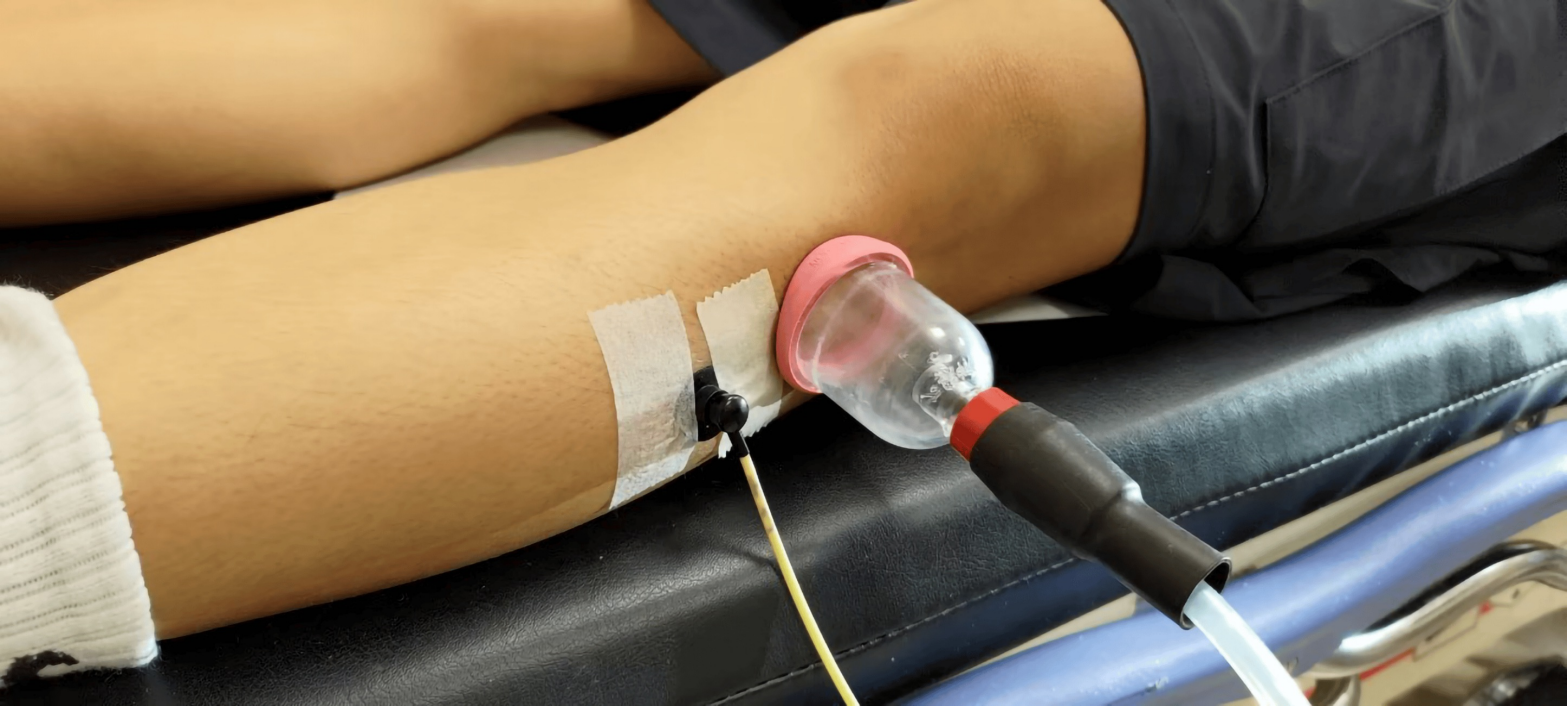
Illustration laser Doppler flowmeter monitoring SBF. Laser Doppler probes are installed 3 cm around negative-pressure treatments. Skin blood flux data recorded by a laser Doppler probe were used to monitor SBF changes for NPT. SBF, skin blood flow; NPT, negative-pressure therapy.

The suction method created negative pressure in the cup using an electronic negative-pressure device (Medi Pump TC-2000V; Anest Iwata Sparmax Co., Ltd., Taipei, Taiwan). Electronic negative-pressure devices do not usually require bloodletting, which makes them safer [39]. A cup with an inner diameter of 45 mm and an outer diameter of 53 mm was used for the cupping therapy. Each side of the cup rim had a width of 4 mm; therefore, the cup rim did not cause pain. It is commonly used in clinical practices. Scar tissue was continuously measured using a laser Doppler probe for 3 min to establish baseline values. Cupping therapy in one of the three protocols was applied at -105, -125, or -145 mmHg for 10 min, followed by scar tissue measurements at the same location for 3 min. The SBF records are presented in Figure 2.

Because the participants had scars at different locations, they were asked to arrive at the study and be placed in a supine position on a treatment bed, relaxed, with the elbow in full extension to avoid interference from the height difference between the measurement location and the heart at the SBF [40]. The specific NPT pressure was predetermined and randomly assigned to each participant in the study. The primary treatment site was selected based on the midpoint of scar tissue. The attending physician subjectively identified the primary scar site and confirmed it via palpation. Cupping therapy in one of the three protocols was applied to the primary site of the scar using one of the three protocols, and the baseline value was measured using a laser Doppler probe. In the NPT, the laser Doppler probe was set at 3 cm around the cup [41]. After treatment, the laser Doppler probe was placed again on the primary site of the scar to record the SBF.

Data analysis

**Figure 3:**
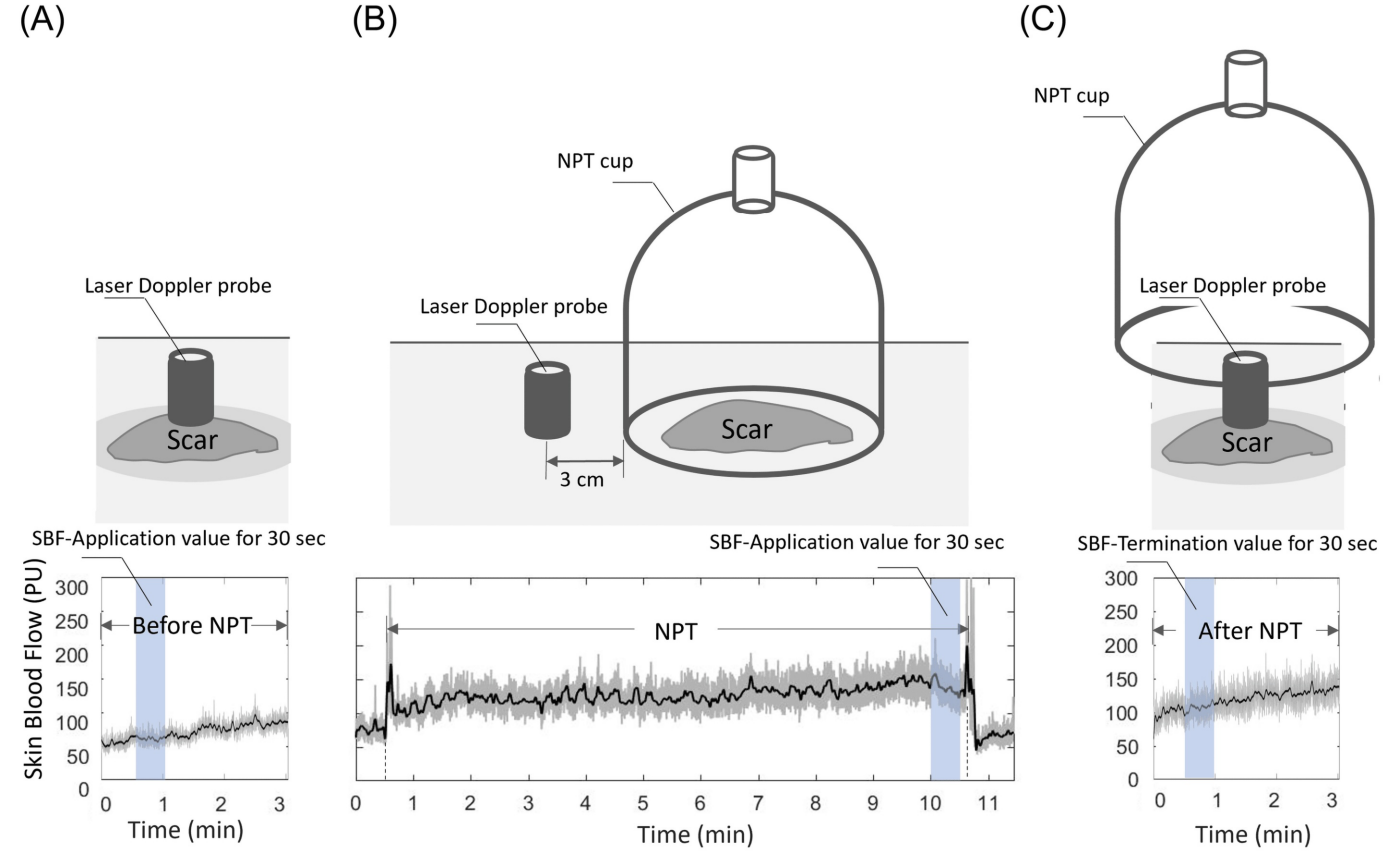
Illustrations of the evaluation standard, 30-second average SBF value in the NPT. (A) SBF-Baseline values were defined as an average of selected phase calculation values from 30 sec to 1 min as an evaluation criterion; (B) SBF-Application values were defined as records of the calculated NPT values between 10 and 10.5 min; (C) SBF-Termination values were defined as SBF after NPT recorded from 30 sec to 1 min. SBF, skin blood flow; SBF-Baseline, SBF measured before treatment; SBF-Application, SBF measured during treatment; SBF-Termination, SBF measured after treatment, PU, perfusion units; NPT, negative-pressure therapy. These illustrations were created by the authors of this paper.

As an evaluation criterion, SBF measured before treatment (SBF-Baseline) values were defined as the average of the selected phase calculation values from 30 sec to 1 min. SBF measured during treatment (SBF-Application) values were defined as records of the calculated NPT values between 10 min and 10.5 minutes. SBF measured after treatment (SBF-Termination) values were defined as SBF after the NPT was recorded at 30 sec to 1 min. In Figures 3A-3C, we show SBF-Baseline value, SBF-Application value, and SBF-Termination value.

Statistical analysis

All values are expressed as mean ± standard error (SE). One-way ANOVA with Fisher's least significant difference (LSD) post hoc test was used for pairwise comparisons of the three NPT dosages (-105, -125, and -145 mmHg) under each protocol phase (SBF-Baseline, SBF-Application, and SBF-Termination). Furthermore, Pearson product-moment correlation analysis was used to determine the correlations between the SBF-Baseline, SBF-Application, and SBF-Termination factors. The significance level was set at P < 0.05. Statistical analysis was performed using the Statistical Package for Social Science (SPSS), version 20 (SPSS Inc., Chicago, IL, USA).

## Results

The average age of all 36 subjects in this study was 38.9 ± 19.0 years. The body mass index (BMI) was 22.9 ± 3.6 kg/m^2. The average duration of injury until the visit for treatment was 22.7 ± 18.9 weeks. All participants were Asian.

The final study included 36 subjects from Asia University Hospital, divided into three groups: 11 in the -105 mmHg group, 13 in the -125 mmHg group, and 12 in the -145 mmHg group. The average ages of the three groups in this study were 35.1 ± 11.5 years in the -105 mmHg dosage group, 39.6 ± 22.3 years in the -125 mmHg dosage group, and 50.8 ± 17.4 years in the -145 mmHg dosage group. BMI showed 22.9 ± 3.8 kg/m^2 in the -105 mmHg dosage group, 21.6 ± 3.0 kg/m^2 in the -125 mmHg dosage group, and 24.0 ± 4.2 kg/m^2 in the -145 mmHg dosage group. No significant differences were observed in any of the parameters between the dosage groups in terms of their demographics. The trial account sample size followed other research [42]. This study used block randomization based on the order of entry into the trial to determine treatment dosages. The NPT dosage was constant throughout the treatment period. According to the randomization schedule, each block was sequentially numbered and placed in a confidential envelope. The participants were assigned a number and received the corresponding NPT dose. The patients and outcome assessors were blinded to the allocation. No important harm or unintended effects on the subjects were observed during the NPT process.

This study analyzed different types of scars caused by surgeries, such as open wound suturing or surgeries to treat conditions such as tumors. This study also examined abrasions resulting from falls or traffic collisions. Hydrothermal burns were the only type of burns considered in this study. Scarring occurs only in cases of allergic reactions to insect bites. The scar information of the subjects regarding the NTP magnitude is presented in Table 1.

**Table 1:** Scar information regarding the NTP dosage. Eleven subjects were used for -105 mmHg NPT dosage. Thirteen subjects were assigned to the -105 mmHg NPT dosage group. Twelve subjects were used for -105 mmHg NPT dosages. n was the number of participants. NPT, negative-pressure therapy.

Division	Category	Magnitude					
		-105 mmHg (n=11 subjects)		-125 mmHg (n=13 subjects)		-145 mmHg (n=12 subjects)	
Injury type	Surgeries	63.6%	n=7	61.5%	n=8	58.4%	n=7
	Abrasions	27.3%	n=3	30.7%	n=4	8.3	n=1
	Burn	9.1%	n=1	7.6%	n=1	25.0%	n=3
	Insect bite	0.0%	n=0	0.0%	n=0	8.3%	n=1
Injury location	Torso	18.2%	n=2	15.4%	n=2	8.3%	n=1
	Upper limbs	36.4%	n=4	46.1%	n=6	75.0%	n=9
	Lower limbs	45.5%	n=5	38.5%	n=5	16.7%	n=2

**Figure 4:**
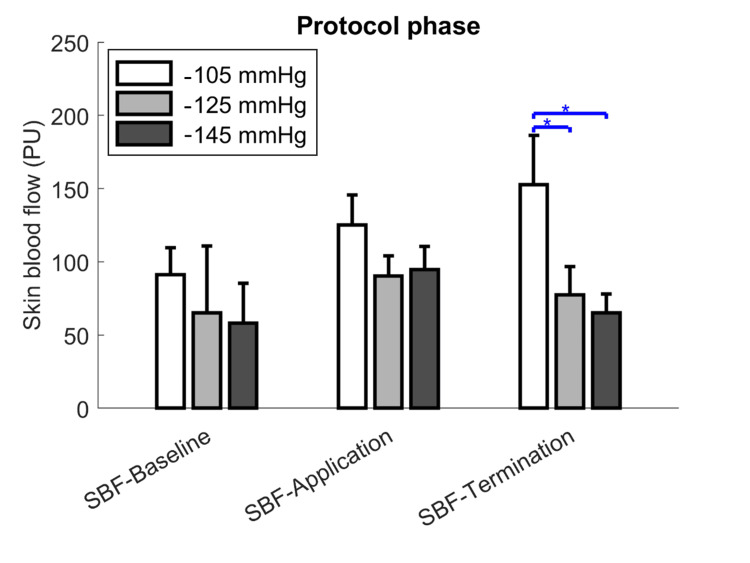
Changes in skin blood flow during NPT in three phases with different dosages. NPT, negative-pressure therapy; PU, perfusion units; SBF, skin blood flow; SBF-Baseline, SBF measured before treatment; SBF-Application, SBF measured during treatment; SBF-Termination, SBF measured after treatment. * P* *< 0.05.

Regarding the effect of SBF-Termination, one-way ANOVA showed that -105 mmHg was greater than other NPT dosages in two significant differences: (1) -105 mmHg and -125 mmHg (152.7 ± 33.7 vs. 77.5 ± 19.2 perfusion units (PU), P = 0.026); and (2) -105 mmHg and -145 mmHg (152.7 ± 33.7 vs. 65.2 ± 12.7 PU, P = 0.012) (Table 2, Figure 4).

**Table 2:** Comparison of SBF response changes using ANOVA. Data are shown as mean ± standard error. SBF, skin blood flow; PU, perfusion units; SBF-Baseline, SBF measured before treatment; SBF-Application, SBF measured during treatment; SBF-Termination, SBF measured after treatment; LSD: least significant difference; * P < 0.05.

Factor	Dosage			One-way ANOVA	Fisher LSD post hoc		
	-105 mmHg (mean±SE)	-125 mmHg (mean±SE)	-145 mmHg (mean±SE)	P value	-105 mmHg vs.-125 mmHg	-105 mmHg vs.-145 mmHg	-125 mmHg vs.-145 mmHg
SBF-Baseline value (PU)	91.2±16.6	65.0±45.8	58.1±27.3	0.181	0.155	0.080	0.697
SBF-Application value (PU)	125.0±20.6	90.3±13.8	94.8±15.6	0.299	0.149	0.216	0.845
SBF-Termination value (PU)	152.7±33.7	77.5±19.2	65.2±12.7	0.025*	0.026*	0.012*	0.698

**Figure 5:**
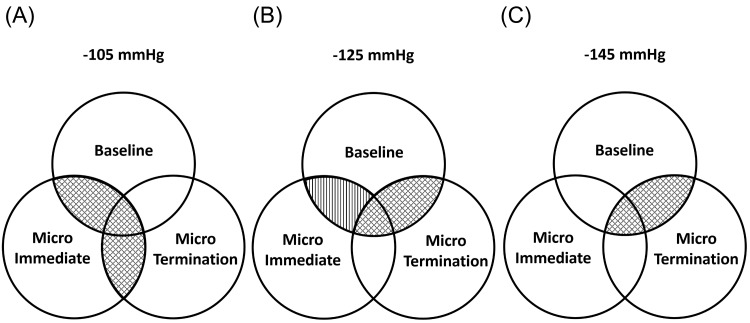
Correlation between SBF-Baseline, SBF-Application, and SBF-Termination in different negative-pressure therapy dosages. Cupping therapy in three protocols was applied at (A) -105 mmHg, (B) -125 mmHg, and (C) -145 mmHg; ▧ Parallel line indicates a significant correlation (P < 0.05). ▩ Cross-line indicates a significant correlation (P < 0.01). SBF, skin blood flow; SBF-Baseline, SBF measured before treatment; SBF-Application, SBF measured during treatment; SBF-Termination, SBF measured after treatment.

Correlation analysis was found to have a greater influence on the SBF-Application and SBF-Termination values. SBF-Baseline and SBF-Application were strongly correlated at -105 mmHg (R = 0.734) (Table 3, Figure 5A) and moderately correlated at -125 mmHg (R = 0.626) (Figure 5B). Furthermore, the baseline relationship with SBF-Termination revealed a strong correlation at -125 mmHg (R = 0.803) and -145 mmHg (R = 0.842) (Figure 5C). The SBF-Application relationship with SBF-Termination showed a strong correlation at a dosage of -105 mmHg (R = 0.890).

**Table 3:** Correlation between different NPT dosages. As an evaluation criterion of NPT, the SBF value were defined three sections including SBF was measured before treatment (SBF-Baseline), during treatment (SBF-Application), and after treatment (SBF-Termination). SBF, skin blood flow; NPT, negative pressure therapy. *, P < 0.05; **, P < 0.01.

Parameter	-105 mmHg		-125 mmHg		-145 mmHg	
	Correlation (R)	P value	Correlation (R)	P value	Correlation (R)	P value
SBF-Baseline vs. SBF-Application	0.734	0.010*	0.626	0.022*	0.381	0.222
SBF-Baseline vs. SBF-Termination	0.531	0.093	0.803	0.001**	0.842	0.001**
SBF-Application vs. SBF-Termination	0.890	0.000**	0.235	0.440	0.281	0.377

## Discussion

This study has several major implications. First, NPT increased the scar SBF. Second, a lower NPT significantly increased SBF compared with a higher NPT, supporting the hypothesis that a suitable NPT would have a greater effect on scar improvement. Third, this study also found a correlation between SBF-Application and SBF-Termination in lower NPT, suggesting a suitable NPT for improving the environment for increased SBF.

Our study revealed that NPT resulted in an increasing trend in scar SBF at each dosage. In this study, SBF was used as a clinical tool to assess various patients and the intensity of the applied mechanical stress. In our study, the SBF-Application showed 100 to 125 perfusion units (PU), which was four to five times higher than the average baseline perfusion. This finding aligns with a previous study indicating that NPT can enhance SBF [43]. NPT may enhance blood flow to the scar tissue. This mechanism of enhanced SBF is thought to be due to the induced dilation of capillaries [44].

NPT could mechanically augment the tensile stresses applied to capillaries to stimulate the endothelium. Capillaries are rapidly dilated as nitric oxide (NO) enters the cells and causes vasodilation, leading to localized hyperemia and rapid NO entry [21]. Localized hyperemia showed an enhanced SBF. According to a previous study, locally enhanced SBF can increase tissue oxygen saturation and red blood cell velocity [18]. In response, the dermis increases oxygen flux from the capillaries to the dermis, and more oxygen diffuses into the dermis [45]. Specifically, increased tissue oxygen levels can improve the hypoxic environment [46]. Preventing scarring as a blockage leads to capillary formation due to a lack of oxygen [22]. This is conducive to effective scar healing while minimizing the risk of damage to similar endothelial cells, fibroblasts, and adverse angiogenesis [47]. Thus, NPT may promote scar healing by influencing SBF, suggesting that it may increase scar healing by influencing SBF.

In this study, the dosage of -105 mmHg showed a significantly higher SBF than both -125 mmHg and -145 mmHg, which means that a higher level of NPT did not result in a higher SBF increase. This contradicts the findings of previous studies, which showed that a higher NPT level results in a higher SBF [48]. However, our results are similar to those of other NPT studies [49]. In this study, a higher NPT level did not result in a higher SBF increase. Based on a comparison of both studies, this could be due to the NPT caused by cupping suction. Although the 4 mm cup rim did not cause pain to the participant, the soft tissues directly under the rim were compressed by shear force, leading to capillary blockage in the facial SBF [37, 50]. This decrease in SBF at higher negative pressures may result from capillary compression caused by excessive tensile and shear forces. The increased mechanical stress could temporarily block capillary perfusion, counteracting the expected hyperemic response.

Overall, in this study, the effectiveness of the NPT dosage in increasing SBF might not be enough to overcome the shear force blocking the capillary, which negates the SBF benefit of a higher dosage. There was a strong correlation between SBF-Application and SBF-Termination only in studies involving the -105 mmHg group. In this study, the SBF-Application parameter referred to the immediate SBF in the NPT was used. NPT generates low pressure inside the device, causing a pressure difference for mechanical augmentation and tensile stress, resulting in increased SBF [21,51]. All dosages of NPT in this study showed improvements in SBF-Application values at each dosage, which could support this perspective.

**Figure 6:**
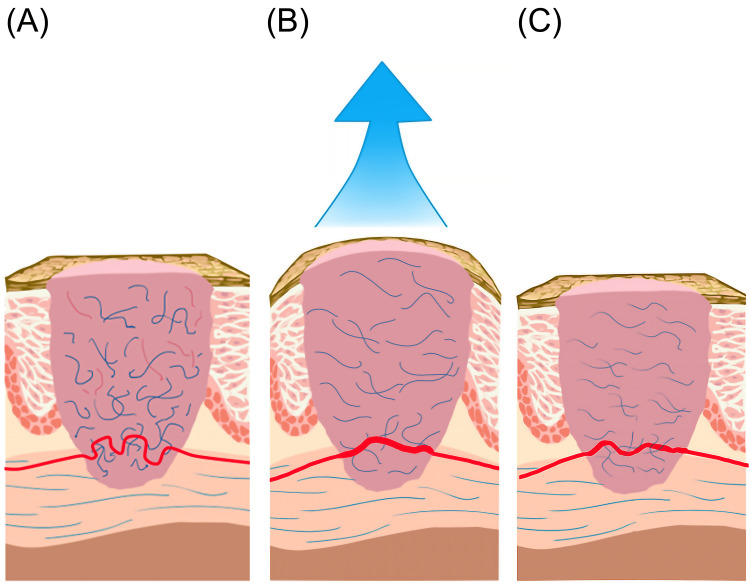
Illustrations of negative-pressure therapy for improving the scar environment. (A) Scar base state, disorganized collagen fibers can compress the capillaries; (B) SBF-Application, negative-pressure treatment increases the space between collagen fibers and relieves compression; (C) SBF-Termination, the collagen fibers are reorganized, which can help to lessen the pressure on the capillaries. SBF, skin blood flow; SBF-Application, SBF measured during treatment; SBF-Termination, SBF measured after treatment. These illustrations were created by the authors of this paper.

The NPT creates a pressure differential on the skin surface [21]. The pressure difference caused by NPT stretches fibrin [52], dilating the scar tissue to release capillary compression and increase blood flow [22]. The results are shown in Figures 6A, 6B.

In this study, SBF-Termination refers to the SBF state after the NPT. If NPT helps align collagen fibers, it reduces blockages in collagen and elastic fibers, improving the capillary environment and increasing SBF-Termination [24]. In this study, after NPT, the SBF-Termination in the -125 and -145 mmHg groups decreased to close to the baseline of NPT, but the SBF in the -105 mmHg group continued to increase. This likely indicates that the capillary environment returned to that before NPT and led to capillary compression by collagen and fiber again, decreasing SBF from SBF-Application to SBF-Termination [8]. Consequently, there was a decline in the improvement in SBF, as shown in Figure 6C.

Based on these results, we observed that NPT at a dosage of -105 mmHg displayed a greater potential for scar healing. In this study, a low dosage of -105 mmHg resulted in significantly higher SBF-Termination. Scarring typically occurs on the superficial skin. Therefore, higher NPT doses affect relatively deep soft tissues [53]. We inferred that excessive NPT results in tiny tears in the scar tissue and prevents collagen and elastic fibers from reorganizing. As a result, it may explain why the lower dosage (-105 mmHg) reduces the obstruction of collagen and elastic fibers, allowing for an increase in SBF after treatment. In contrast, higher dosages (-125 and -145 mmHg) did not increase SBF-Termination.

This study had two main limitations. First, we focused only on the immediate effects of NPT. Scar recovery is a long-term process that typically takes approximately one to two years for scars to heal after an injury [37]. We may not be able to generalize our results to long-term characteristics, such as efficacy after a week. This preliminary study aimed to establish a scar treatment regimen using different NPT doses. Future follow-up studies will be conducted to precisely track the efficacy of the treatment in patients with long-term scars to improve clinical benefits. Second, the participants in this study had inconsistent scar locations based on random invitations during the recruitment process. Different scar locations may have caused the baseline SBF to differ [54]. In future studies, recruited subjects may undergo NPT in specific areas, such as the legs and hands.

## Conclusions

This is the first study to evaluate SBF in scars using different NPT doses. This study provides the first evidence that -105 mmHg NPT increases SBF more than -125 or -145 mmHg. This study found that only the -105 mmHg group demonstrated a consistent relationship between SBF during and after treatment, suggesting a more sustained perfusion effect. Furthermore, these results highlight the importance of dosage selection in NPT and indicate that -105 mmHg may be a promising setting for optimizing the perfusion response in scar tissue.
